# Recent Advances in Microfluidics-Based Electrochemical Sensors for Foodborne Pathogen Detection

**DOI:** 10.3390/bios13020246

**Published:** 2023-02-09

**Authors:** Madhusudan B. Kulkarni, Narasimha H. Ayachit, Tejraj M. Aminabhavi

**Affiliations:** 1 Renalyx Healthcare Systems (P) Limited, Bengaluru 560004, Karnataka, India; 2 School of Electronics and Communication Engineering, KLE Technological University, Hubballi 580031, Karnataka, India; 3 School of Advanced Sciences, KLE Technological University, Hubballi 580031, Karnataka, India

**Keywords:** electrochemical, microfluidics, sensors, food safety, nanotechnology, pathogens

## Abstract

Using pathogen-infected food that can be unhygienic can result in severe diseases and an increase in mortality rate among humans. This may arise as a serious emergency problem if not appropriately restricted at this point of time. Thus, food science researchers are concerned with precaution, prevention, perception, and immunity to pathogenic bacteria. Expensive, elongated assessment time and the need for skilled personnel are some of the shortcomings of the existing conventional methods. Developing and investigating a rapid, low-cost, handy, miniature, and effective detection technology for pathogens is indispensable. In recent times, there has been a significant scope of interest for microfluidics-based three-electrode potentiostat sensing platforms, which have been extensively used for sustainable food safety exploration because of their progressively high selectivity and sensitivity. Meticulously, scholars have made noteworthy revolutions in signal enrichment tactics, measurable devices, and portable tools, which can be used as an allusion to food safety investigation. Additionally, a device for this purpose must incorporate simplistic working conditions, automation, and miniaturization. In order to meet the critical needs of food safety for on-site detection of pathogens, point-of-care testing (POCT) has to be introduced and integrated with microfluidic technology and electrochemical biosensors. This review critically discusses the recent literature, classification, difficulties, applications, and future directions of microfluidics-based electrochemical sensors for screening and detecting foodborne pathogens.

## 1. Introduction

In recent times, microfluidics technology has gained substantial attention among researchers and scientists, especially in electrochemistry and biochemistry studies, for imitating the traditional benchmark laboratory instruments on a miniaturized chip-based system [1,2,3]. Microfluidics is applied almost in all fields, such as biomedical, electrochemical, pharmaceutical, clinical, and biochemical domains. It offers advantages like minimum sample volume, fast response, precision, multiplex operation, and rapid assessment [4,5,6]. These properties convey significant resources to electrochemical and biochemical facets. Research in microfluidics has made noteworthy advancements over the recent decades and has grown in popularity because of the vital characteristic benefits such as portability, versatile design, minimal reagents, the potential for simultaneous process, and easy connection to a smartphone for data access and storage on the cloud. Microfluidics is an open platform for the automation, integration, and miniaturization of devices highly suitable for electrochemical, biomedical, and biochemical applications. Further, the microfluidic technique plays a vital role as the on-chip methdo of detection of viruses, pathogens, diseases, and bacteria in nephrology, neurology, cardiology, ophthalmology, and oncology [7,8,9,10,11,12,13].

**Figure 1:**
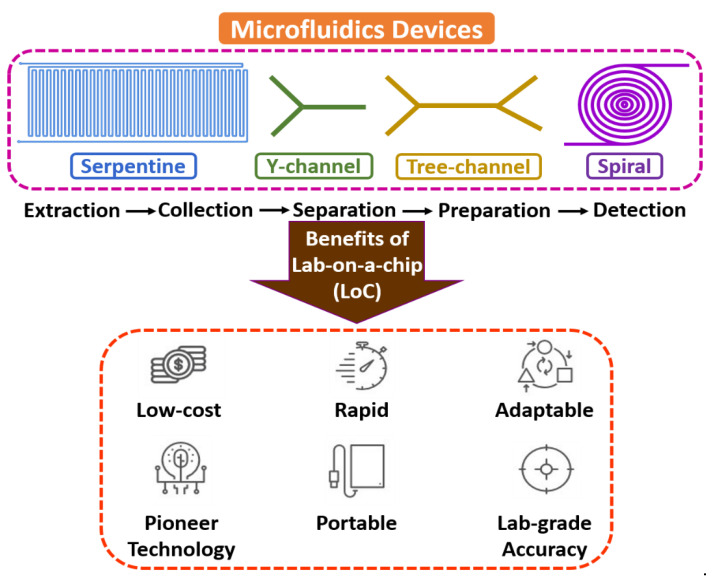
Fundamentals of microfluidic devices.

Microfluidics is an interdisciplinary field with a broad overview of sample extraction, collection, separation, preparation, manipulation, coordination, and detection at a microscale environment [14,15,16]. The flow mechanism is generally led by surface tension, capillary, Van der Waals forces, and electrostatic processes. It is the backbone of the biological microelectromechanical system (BioMEMS), the micro total analysis system (µTAS), and lab-on-a-chip (LoC) domains, as most of the electrochemical assessments include fluid devolution and reaction for real-time sensing applications. Microfluidic technology’s electrochemical and biochemical reactions are typically faster due to few reagents and minimum volume [17,18,19]. Figure 1 shows the fundamentals of microfluidic devices.

Microfluidic devices are developed to manipulate and regulate the fluid management within the microcapillary, reducing the overall volume of reagents and apparent for efficiency and effectiveness of mass and thermal aspects because of their better surface-to-volume ratio. Erickson et al. [20] described a study on an integrated microsystem that was used for cytometry and cell management. Manz et al. [21] made a review of µTAS that extended over the expansion history and concept of miniaturization and fabrication of microfluidic systems, typically for sample extraction. Kulkarni et al. [22] discussed nanomaterials’ synthesis on a miniaturized microreactor and compared conventional approaches. Fair et al. [23] demonstrated the operation of blood cells in a micro/nano-environment and introduced high throughput for separating blood cells with plasma. Kulkarni et al. [24] reported a continuous-flow-based µ-PCR system with an integrated approach used for biomedical applications such as cell culture [25], nucleic acid [26], albumin-to-creatinine [27], and saliva [28].

**Figure 2:**
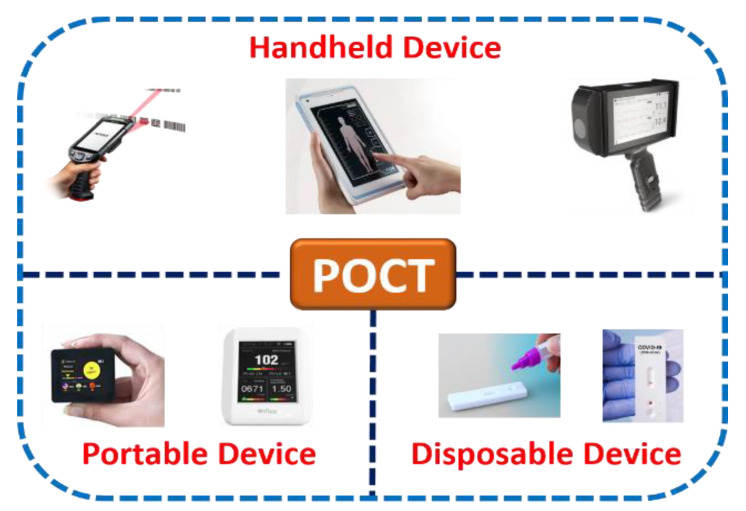
Classification of point-of-care-testing (POCT) microdevices.

Microfluidics combines science involving fluidic activities on a miniature platform and technology involving the design, calibration, optimization, execution, and fabrication of such microdevices for abundant point-of-care applications. Figure 2 shows the classification of various point-of-care-testing (POCT) microdevices. Further, considering numerous additional advantages of microscale technology over benchtop laboratory instruments due to their high flexibility and capability to produce new building blocks, it is indispensable to establish and conceptualize POCT devices that can be suitable for medical diagnosis. POCT is a primary diagnostic assay that lets unskilled persons or non-physicians to provide medical tests with affordable, rapid, and limited resources near patients [29,30,31].

Nanomaterials are presently experiencing rapid development because of their distinctive optical, thermal, electrical, physical, and mechanical qualities that have potential applications in biosensors, electrochemical sensors, catalysis, magnetic data storage, structural components, nano-electronics, and biomaterials. Usually, nanomaterials are made up of tiny particles smaller than 100 nm [32]. The term nanotechnology refers to materials that are several hundred nanometers or sub-nanometer in size. Virtually, it is impossible to execute any application without nanomaterials in science and technology. It is anticipated that the effective utilization of nanomaterials will improve the functionality of biomolecular electrical devices with high sensitivities and detection limitations. Further, it is being investigated how nanoparticles, nanowires, nanotubes, nanorods, graphene, and MXenes can be used in biosensor diagnostic applications [33,34,35]. Smart biosensors that can detect minute concentrations of the desired analyte are getting developed as a result of advancements in the characteristics of nanomaterials and their dimensions at the nanoscale level. Typically, nanomaterials are utilized as a transducing material, which is crucial for developing biosensors [36].

In recent years, biosensors have opened new horizons by emphasizing biological, biomedical, and electrochemical domains to assist healthcare, food safety, agriculture, and environmental monitoring [37,38,39]. Biosensors are acquiring the attention of researchers, academicians, and industrialists due to their excellent capability to recognize an electrochemical occurrence on a transducing module using a signal relative to a concentrated solution to compute a chemical process. Trends in microfluidic technology have enabled the design of miniaturized biosensors to regulate, coordinate, and alter micro/nano-volumes of sample fluid through capillary microchannel dimensions ranging from 1 to 100 µm. Biosensors can have different sizes, structures, and bioelectrodes that can sense and assess viruses, diseases, and pathogens. There are different biosensors based on their applications, such as electrochemical, biological, and biomedical sensors [40]. Among these, electrochemical sensors are widely used for various food safety, pharmaceutical, environmental, and agricultural applications [41,42,43].

One of the common sensing methods is the electrochemical nanobiosensor, which converts biological events into electrical impulses [44,45]. Here, an electrode is a fundamental component used as a stable foundation for immobilizing biomolecules and the transportation of electrons [46]. Electrochemical sensors are microdevices that provide data about the conformation of a system in real time by pairing a chemically discriminating layer to a transducer [47]. In this manner, the chemical energy of the selective interface between the chemical species and the sensor is transduced into an analytically advantageous signal for further analysis. Electrochemical sensors are the biggest and oldest group of chemical sensors because the techniques and equipment needed are so straightforward. As these sensors are simple to automate and integrate into smaller spaces without sacrificing analytical capabilities, they are very popular in recent times. Different families of electrochemical sensors can be identified depending on the electrical magnitude employed to transmit the recognition event [48]. In order to achieve excellent performance in terms of analytical sensitivity, nanomaterials with a wide surface area and synergic effects are made possible by boosting loading capacity and the mass transport of reactants. In recent times, electrochemically driven biosensing approaches have been familiarized for easy and portable analytical devices for on-site detection [49]. Further, this trend can realistically replace the conventional lab-based tools produced by the prominent in vitro diagnosis companies that allege susceptible measurement of automation and analytes [40,50,51,52].

Pathogens are contagious mediators, including microorganisms that can cause diseases, such as protozoans, fungi, prions, viruses, and bacteria in the human body [43,53,54]. Foodborne pathogens can enter the body via several ways of infection [55]. Here, the primary causes of food safety issues are foodborne diseases transported by ingesting food contaminated with germs [56]. Pathogenic bacteria that produce foodborne infections are *Salmonella* spp., *Escherichia coli* (*E. coli*), *Staphylococcus aureus* (*S. aureus*), *Shiga-toxin-producing Escherichia coli*, *Listeria monocytogenes* (Lm), *Campylobacter* spp., *Enterobacter sakazakii*, and *Clostridium botulinum* [57,58,59,60]. The primary indicators of foodborne pathogens in humans are food poisoning, dysentery, diarrhea, and even death. Bacterial infections cause a staggeringly high number of deaths each year; 13 million deaths worldwide are expected by the year 2050. Over 91% of foodborne outbreaks, especially in the USA, are caused by the most commonly reported foodborne pathogenic bacteria [61]. Consequently, it is important to recognize and detect foodborne pathogenic bacteria. Further, traditional culturing techniques, nucleic-acid-based techniques, and immunological analyses such as PCR-ELISA are the main approaches for identifying foodborne pathogenic bacteria [62]. However, these methods lack the necessary advantages for point-of-care applications since they are time-consuming, expensive, require a specific bulky device, and are unstable [31,63,64].

Foodborne pathogens are routinely detected using time-consuming, tedious methods such as nonselective and selective enrichment culture, plate separation, pure stages, biochemical reaction, and serological identification. The conventional methods are unable to encounter the necessity of food safety supervision and rapid diagnosis in detecting food pathogens. In recent times, detection techniques were established with the development of food technology, such as detecting certain bacteria with an automatic identification system and POC technology. However, these methods still have a few limitations such as requirement of purifying cultures and enriching foodborne bacteria. Furthermore, there may be more than one microorganism and pathogen in food, hence, it is desirable that one platform detects multiple target microorganisms and pathogens simultaneously. This is possible with microfluidics-based electrochemical biosensors for rapid detection of foodborne diseases.

The present article critically discusses the principle, classification, and recent advances of microfluidics-based electrochemical sensors for sustainable food safety and foodborne pathogens and compares them with the state of the art. Furthermore, challenges and limitations involved in microfluidic electrochemical biosensors for commercialization as a product are considered. Finally, the future directions of microfluidics-based electrochemical sensors used to identify foodborne pathogens are discussed.

## 2. Recognition of Elements of Biosensors and Electrochemical Biosensors

**Figure 3:**
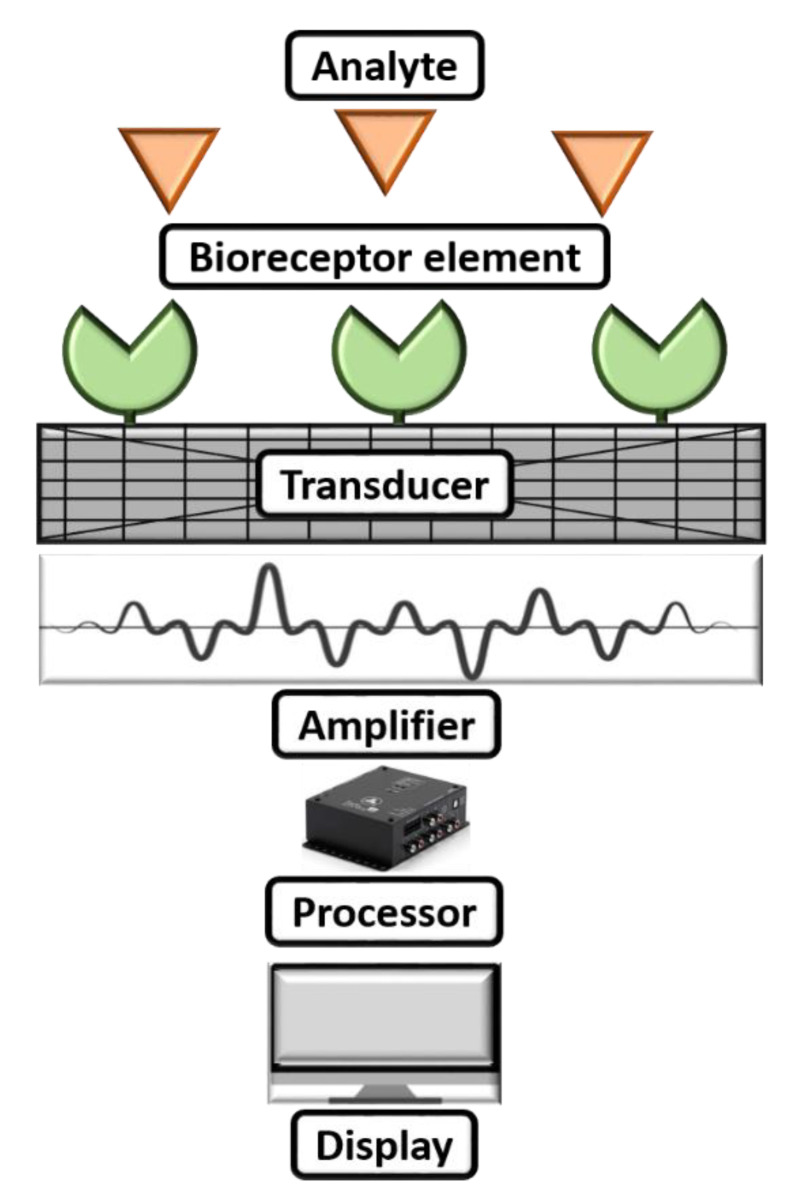
Working mechanism of a biosensor.

A biosensor can be commonly described as a diagnostic microdevice that translates a biological reaction into an assessable and transmutable signal [65]. Figure 3 shows a simple working mechanism of a biosensor. Generally, biosensors are small microdevices with an electrode modified with a bioreceptor element consisting of an electronic reader responsible for recording, collaborating, and sensing of physiological constraints of biochemical components [66]. Furthermore, they can be employed to find viruses in food, water, the environment, and farming. The working principle of biosensors involves various parameters: (i) analyte, (ii) bioreceptor element, (iii) transducer, (iv) electrical signal, and (v) display. Herein, the synthesized nanomaterial is coated after the analyte to modify the sensing electrode to boost the sensing parameters on the biosensor. The electrode will be introduced into a buffer electrolyte, and its components, such as glucose, ammonia, alcohol, and lactose, will be recognized. When analyte and bioreceptor components interact, a signal is produced. The transducer converts this signal into an amplified electrical signal, indicating the existence of a biochemical objective. Here, the optical or electrical signals produced by the transducers, which can be connected to the cloud for simple data access, are proportional to the analyte–bioreceptor interactions. Finally, the results can be in the form of graphical, tabular, or mathematical studies.

**Figure 4:**
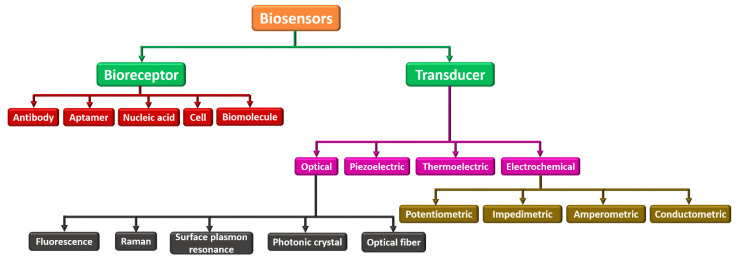
Classification of various biosensors based on their bioreceptor element and transducing component.

Biosensors can be categorized into many kinds based on their analytes or components to be sensed as bioreceptor elements and transducing components. Figure 4 demonstrates different biosensors categorized based on the bioreceptor element and transducing component. Parametric sensors, such as a microphone and strain gauge, are active sensors that require an external power source. In contrast to the photodiode and piezoelectric sensors, passive sensors do not need any external power. Utilizing the specific type of signal detection, these biosensors are categorized into (i) physical, (ii) thermal, (iii) biological, and (iv) chemical. These are widely employed in electrochemical, biochemical, and BioMEMS domains [67,68,69]. Based on bioreceptor elements, biosensors can be either catalytic or noncatalytic. Here, the chemical reaction can happen between the solution and bioreceptor in a catalytic biosensor. For example, whole cells, tissues, enzymes, the immune system, and bacteria fall under this category. In a noncatalytic biosensor, the solution is irrevocably combined with the bioreceptor with no new chemical reaction. For example, nucleic acid, aptamer, cell receptors, and antibodies fall under this group [70,71].

Further, based on the transducer, they can be categorized as (i) optical, (ii) piezoelectric, (iii) thermoelectric, and (iv) electrochemical. An optical biosensor, in this context, is a tiny analytical tool made up of an optical transducer and a bioreceptor component. The optical biosensor provides a signal that is proportional to the concentration of the additional investigational reagents. Usually, optical biosensors are further classified as surface plasmon resonance, photonic crystal, and optical fiber. The piezoelectric biosensor is an analytical device that works on the source of affinity interaction recording. The thermoelectric biosensor works on sensitive technology that reads the bioreceptor’s temperature change. Finally, an electrochemical biosensor works on the principle that electrodes interpret a chemical reaction into an electrical signal. Electrochemical biosensors are widely used to sense several bioanalytes in the human body, such as blood ketones, cholesterol, glucose, uric acid, cells, tissues, urea, lactate, nucleic acid, and hemoglobin [34,72,73].

At the moment, electrochemical sensing technology has a wide range of applications because of their unique benefits like low detection limit, sensitivity, and simple process. Compared with existing conventional analytical techniques, microfluidic technology has advantages in real time that can make mutual interactions with pathogens and analyze the variations that occur at every instant of the process. Additionally, it is fast, as the process takes only 5–10 min, and a big number of reactions can be determined in a short time. It detects specific and nonspecific particles in the reagents. Finally, it is simple, as big particles do not require to be labeled. The evolving electrochemical technique has been developed and used for food safety; the analysis can be done in a much shorter time, with high selectivity and sensitivity that can be comparable to that of the traditional approaches, which makes the idea of rapid detection of foodborne pathogens possible in a real-time scenario.

Further, it finds applications in detecting foodborne pathogens and environmental monitoring. Electrochemical sensors are mainly classified into three types: (i) potentiometric, (ii) impedimetric, (iii) amperometric, and (iv) conductometric. Typically, electrochemical sensors work on a three-electrode system: working electrode (WE), reference electrode (RE), and counter electrode (CE). The instrument used for analyzing the biomolecule is known as a potentiostat [74]. Here, the purpose of the reference electrode is to serve as a standard for establishing and controlling the voltage of the working electrode without an admitting current. The reference electrode needs to have a steady electrochemical potential at low current densities. Furthermore, because the reference electrode only passes a very small amount of current, the IR drop between the working and reference electrodes (iRU) is consistently rather low. With the three-electrode system, the reference potential is considerably more stable, and the IR drop across the analyte is compensated. This improves control over the working electrode’s voltage. The most popular laboratory reference electrodes are the Ag/AgCl electrode and the saturated calomel electrode. In the three-electrode design, the counter electrode’s only purpose is to pass all the current necessary to balance the current observed at the working electrode. The counter electrode repeatedly swings to extraordinarily high potentials in order to perform this function [75,76,77].

**Figure 5:**
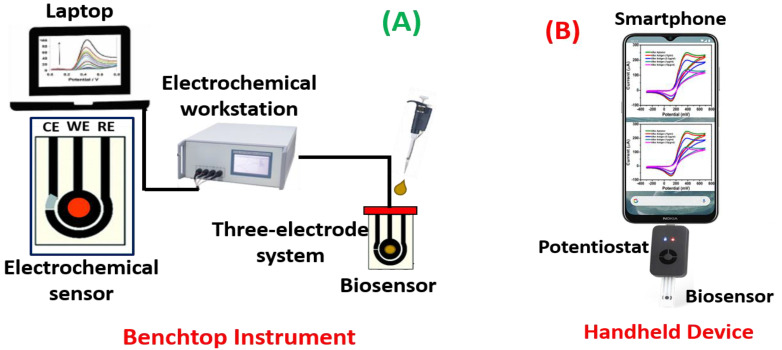
Schematic representation of electrochemical biosensors as (**A**) a benchtop instrument and (**B**) handheld device.

Potentiometric biosensors are used to measure the potential of WE at a constant level concerning the RE. Here, the charge accumulates on the WE because of the interaction between biomolecules and bioreceptor qualified to the RE under zero current. These sensors can detect the current generated by oxidation or reduction using electrochemistry in the electroactive reactant at the WE when a continual voltage is functional to the working electrode relative to the reference electrode [78,79]. Impedimetric biosensors are used to quantify the degree to which an electrochemical reaction affects the impedance between two electrodes [80]. Utilizing these biosensors to track the metabolic activity of living biomolecules is a common process. When a minimum sinusoidal pulse is carried, these sensors can sense electrical impedance generated by electrode contact. Using an impedance analyzer, the in/out-of-phase current response to low-amplitude AC voltage given to the sensor electrode is measured as a function of frequency [81,82]. Amperometrics are used to determine the current with a controlled voltage deviation fed as an input to sense the biomolecules. The primary benefit of these biosensors is the high sensitivity, selectivity, and concurrent identification of several biomolecules [83,84]. Figure 5 illustrates a schematic for electrochemical biosensors as a (A) benchtop instrument and (B) handheld device.

The first-generation glucose oxidase (GOx) biosensor was presented in 1962, and this marked the beginning of the entire field of biosensors [85]. Despite numerous advancements in the generations of biosensors since the 1960s, the GOx sensor is still the most popular among other biosensors. As demonstrated earlier [86], electrochemical biosensors do not suffer from high sensor arrangement complications and price. This is because of their adjacent association with affordable microelectronic circuit production advancements and their simple communication with regular automatic digital read-out and manipulation using a smartphone. The inherent benefits of electrochemical biosensors are their heftiness, ease-to-use, portability, exceptional limits of detection, the minuscule volume of analytes, and the capability to be used in chaotic biological fluids with optically fluorescing and absorbing mixtures. However, few characteristics could have curbed the advent of further revolutionizing the applications constructed on biosensing parameters using an electrochemical concept. The lack of surface topologies that enable sufficient sensitivity and distinctive association of the reaction with the intended biological occurrence has been a problem for electrochemical biosensors. Significant clusters of biosensors, like aptamers, DNA sensors, and antibodies may respond differently depending on the pH and ionic strength of bioanalytes. These problems may be resolved by integrating the next generation of extremely specialized, selective, responsive, and consistent biological and electrochemical sensor arrays that combine solid-state and surface mechanics knowledge with incorporated circuits, bioengineering, and data processing. Thus, it is timely to recapitulate new advancements in this varied field and confer its future direction for developing microfluidics-based electrochemical sensors. Table 1 summarizes different techniques of electrochemical biosensors.

## 3. Electrochemical Sensors for the Detection of Foodborne Pathogens Using Microfluidic Technology

**Figure 6:**
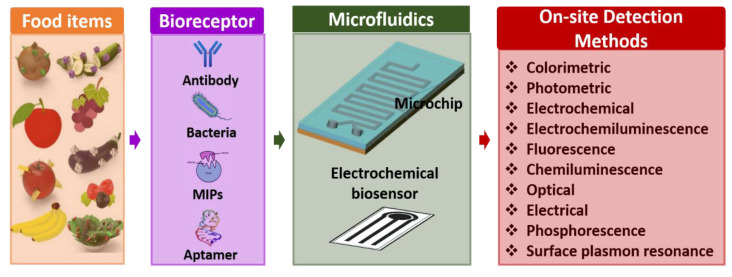
Overview of microfluidics-based electrochemical biosensors for detection of foodborne pathogens.

Microfluidics-based electrochemical sensors are advanced transduction systems for sensing and assessing foodborne pathogens. Usually, microfluidic electrochemical biosensors measure an electrochemical reaction [94]. These enable the conception of small system designs with straightforward instrumentation by directly transforming the inward electrical signal into an electric field [95]. Further, these exhibit generic benefits over analytical workstations: (i) comparable influential sensitivity, (ii) operating potential in murky media, and (iii) potential for miniaturization, which enables analysis of even minuscule volumes. Microfluidics-based electrochemical biosensors are frequently used for identifying pathogenic bacteria in food science. Electrochemical biosensors are an excellent option that can be focused on detecting multiple foodborne pathogenic bacteria such as *Escherichia coli* (*E. coli*), *Lm*, *Salmonella*, *S. aureus*, and other bacteria [96,97]. It is known that only some microfluidics-based electrochemical sensors could sense pathogenic bacteria with a limit of 1 CFU/mL [88]. Here, the sensing electrodes of the electrochemical biosensor are fabricated and modified using unique nanomaterials for better results because of their favorable properties. Figure 6 shows the overview of microfluidics-based electrochemical biosensors for the detection of foodborne pathogens.

As a significant participant in the celiac ecology of mammals, *Escherichia coli* subsidizes the production of vitamin K2 in human beings. However, several harmful strains can lead to the development of specific pathotypes in the urinary tract, and the gastrointestinal tract can bring about local illnesses. There are three common clinical conditions: sepsis, diarrhea, and meningitis. In addition, gastrointestinal diseases constitute a key contributor to morbidity and newborn and early child mortality in South Africa, UK, Asia, and USA. Thus, it is imperative to screen *E. coli,* particularly in food science.

The genus Salmonella is also an essential participant among the domestic Enterobacteriaceae, which includes Salmonella bongori and Salmonella enterica. In general, infections caused by 2500 Salmonella serovars are predominantly linked with tainted food items, normally vegetables, pork, eggs, fresh fruits, pork, and poultry. Table 2 illustrates the electrochemical biosensors for detection of *Escherichia coli* (*E. coli*), *Salmonella*, *Staphylococcus aureus* (*S. aureus*), *Listeria monocytogenes* (Lm), and other bacteria.

**Figure 7:**
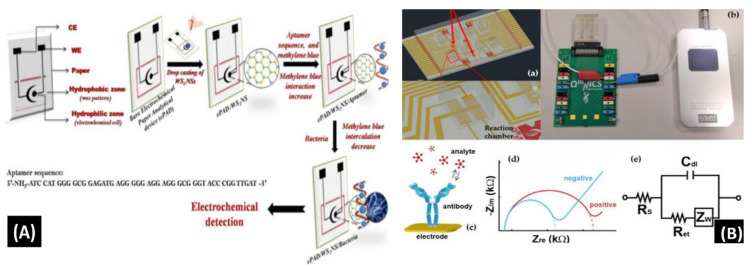

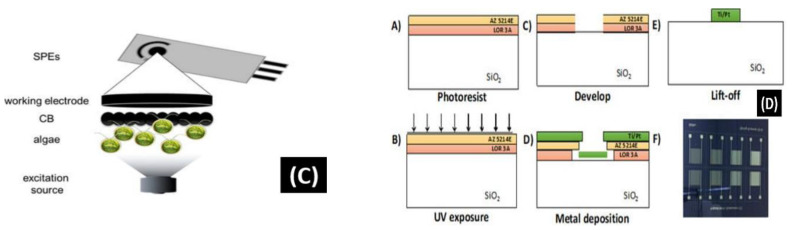
(**A**) Schematic of the screen-printed paper-based aptasensor for the detection of *Listeria monocytogenes* [90]. (**B**) Schematic representation of the LOC device optimized for the detection of GLRaV-3 and GFLV [140]. (**C**) Scheme of the proposed algal/CB-SPE cytosensor [141]. (**D**) Microfabrication procedure for platinum interdigitated electrodes on SiO_2_ wafers [142].

Mishra et al. [90] reported a novel paper-based aptamer that works on an electrochemical sensing platform employed for detecting *Listeria monocytogenes* (Lm). *Listeria* is a renowned causal pathogen for foodborne diseases. The aptasensor has several useful features: it is simple, reliable, disposable, and cost-effective (Figure 7A). The use of an aptamer adds more beneficial features in the biosensor field. Furthermore, the detection and quantification limits of the aptasensor were found to be 10 and 4.5 CFU/mL, respectively, within a range of linearity of approx. 101–108 CFU/mL.

Buja et al. [140] demonstrated the detection of ampelovirus and nepovirus on a microfluidics-based chip. It includes a multichamber design for determining quadruplicate and instantaneous identification of these targets. It can detect the Grapevine fanleaf virus (GFLV) and GLRaV-3 at dilution factors more than 15 times greater than those of ELISA, offering higher sensibility in the identification of these viruses (Figure 7B). Furthermore, this microfluidic platform is simple, fast, miniaturized, and affordable, showing its potential application for large-scale screening assays.

Antonacci et al. [141] discussed an algal cytosensor based on electrochemical computation of bacteria in garbage water, the green photosynthetic alga *Chlamydomonas reihardtii* restrained on carbon black (CB) nanomodified screen-printed microelectrodes. Due to their capacity to detect the oxygen produced by the algae and the current, the CB nanoparticles are used as nanomodifiers, which increases as the number of bacteria exposed to the algae increases (Figure 7C). The sensor was evaluated for detecting *E. coli* in real garbage water samples and reference solutions, with a linear response range of 100 to 2000 CFU/100 mL.

Sidhu et al. [142] illustrated a platinum IDE microelectrode-based aptamer for identifying *Listeria* spp. in hydroponic lettuce growth media. The sensor is a component of a particle or sediment hydroponic lettuce system trap for real-time irrigation water analysis (Figure 7D). The electrochemical behavior was characterized in great detail in *Listeria* spp. DNA presence/absence was followed by calibration in several solutions. The aptasensor showed a 90% recovery rate and could only be used a few times after a quick cleaning.

Although a few conventional approaches for identification of foodborne pathogens are sensitive, most of them are time-consuming, which limits their practical application. Therefore, developing new methods to detect foodborne pathogens is essential, as shown in Figure 7A–D. Microfluidics-based electrochemical biosensing has been sensibly applied for quick determination of pathogens via investigation and development. Electrochemical biosensors based on aptamers or nucleic acid have a low detection limit and high sensitivity; nevertheless, their accuracy and stability should be improved.

## 4. Critical Challenges and Discussions

According to the International Union of Pure and Applied Chemistry (IUPAC) description provided in 1992, a biosensor uses specific biochemical reactions mediated by isolated enzymes, tissues, cells, immunosystems, and organelles to sense chemical compounds generally by thermal, electrical, or optical signals [143]. In the last few decades, enthusiastic research has been conducted to synthesize novel nanostructured materials like CNTs, fullerene, graphene, MXene, and other metal oxides, which can be used for sensing various biomolecules [144,145]. These materials greatly influence the selectivity, sensitivity, stability, and reduction of overpotential. Usually, these nanomaterials are drop-cast and gently coated on the microfluidic electrochemical biosensor’s working electrode. Although there has been a substantial enhancement in the progress of microfluidic electrochemical sensors for detecting foodborne pathogens, a few limitations encumber these applications from being used further [146,147]. Furthermore, the major challenge in the miniaturization of biosensors involves an adequate trade-off between sensor dimensions–signal transduction efficiency and reaction-transport kinetics.

Several requirements must be met in order to build an effective microfluidic electrochemical biosensor for the nonspecialist market:

(i) The biocatalyst must exhibit low variance between assays under typical storage conditions and must be highly selective for the goal of the analysis [148,149].
(ii) The reaction must be unaffected by pH, stirring, and temperature. As a result, samples can be analyzed with nominal pretreatment. Co-immobilizing the composition with the enzyme is preferred [150].
(iii) Over the absorption range of concern, the reaction should be exact, precise, repeatable, and linear without dilution or concentration. Additionally, it must be devoid of noise caused by electrical or other transducers [151,152,153].
(iv) The probe must be small, biocompatible, and free of toxic or allergenic effects if the biosensor is to be utilized for invasive screening in clinical sites. The biosensor should not be susceptible to proteolysis or deactivation either [154,155].
(v) Real-time analysis from the biosensor is preferred for the quick determination of analytes from living samples [156].
(vi) The entire biosensor should be affordable, compact, portable, and used by operators with some ability [157].

Extremely responsive sensing of foodborne pathogens is still an objective chased by many researchers. A minimum volume of samples from the human body, including the skin, intestines, and other organs, can be extracted and detected utilizing highly sensitive biosensors. The real sample could be further diluted before being tested. Furthermore, biosensors with a low detection limit are beneficial for quickly concealing alleged patients. Researchers in the relevant field should propose how to equalize the accuracy and sensitivity of the biosensor because high sensitivity can result in low accuracy. In addition, nonspecific biosensor adsorption in the food composite matrix may result in subpar detection outcomes. Thus, it is unavoidable to continue on monotonous pretreatment of the real biomolecule. Presently, very few biosensors have accomplished commercial success as a product, except electrochemical glucose biosensors and pregnancy tests. Here, the development of microfluidic electrochemical biosensors for foodborne pathogen detection incorporated into low-cost, portable, high-precision, and easy-to-use devices remains a challenge [158,159,160]. Although the sensor’s performance has not yet exceeded the highest sensitivity for foodborne pathogens achieved using multifaceted, expensive, and labor-intensive approaches such as PCR and mass spectrometry, it has high selectivity and quick detection and is economical, which offers significant benefits over prominent detection technologies [94,161].

Here, the utilization of nucleic acids and aptamers as biorecognition elements that keep their long-lasting action is a crucial problem for researchers. The viability of detecting constituents in biosensor applications has been inadequately explored. According to the physical characteristics of the nanomaterial type, the fabrication method and toxicity of functional nanomaterials differ based on the application. As a result, the critical issue raised after the investigation by scientists includes enhancing the stability of biorecognition elements and functional nanomaterials to develop good service life of microfluidics-based biosensors [162,163,164].

**Figure 8:**
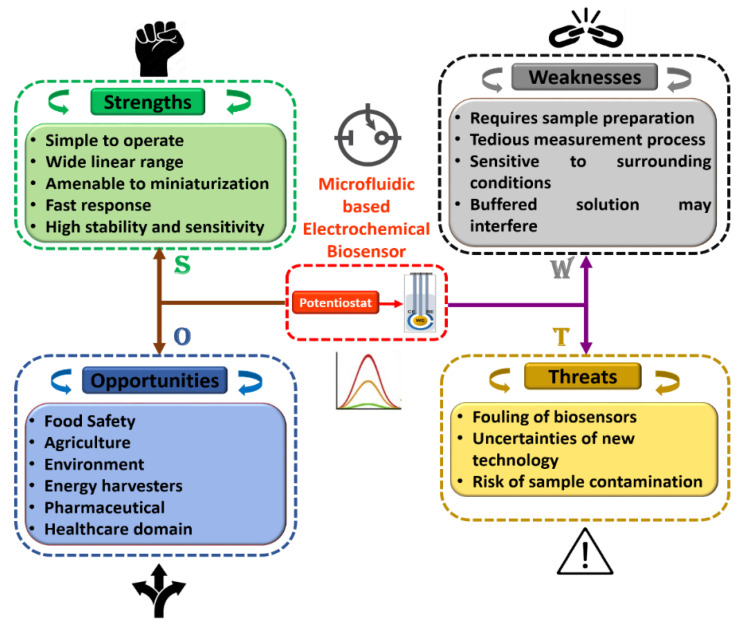
Detailed SWOT analysis of microfluidics-based electrochemical biosensors.

Furthermore, in order to attain a sound understanding of microfluidics-based electrochemical biosensors, a SWOT analysis was plotted as shown in Figure 8. Herein, tactical preparation and premeditated management technique are used to help researchers and scientists to recognize strengths, weaknesses, opportunities, and threats of project planning in any domain of study. Additionally, it can be observed that each discipline has its own room for improvement, which can be achieved with experience and technological progress. We fully believe that these flaws may be overlooked, given the advantages of the integration of this domain of research. Moreover, we believe that the association of microfluidics-based electrochemical sensors with modern engineering in the near future will open up the path for accomplishing intelligent microfluidic electrochemical devices for POCT applications in the field of food science.

## 5. Conclusions and Future Directions

Foodborne pathogenic microorganisms are still relevant and continue to manifest their toxicity. Timely anticipation, precise detection, and profound and fast response in monitoring and sensing pathogens are very important. A microfluidic electrochemical biosensor is a viable and essential analytical microdevice for virus and pathogen investigation. Ideally, an electrochemical biosensor needs the following attributes: (i) The port of the biosensor is ideally designed to have the benefits of vast surface area, ease of use, high specificity, and low cost; (ii) the whole device can be commercialized, automated, integrated, and miniaturized. Even today, only a few biosensors have fulfilled these conditions, and most electrochemical biosensors still have difficulties in commercializing.

Selectivity is one of the significant parameters in deciding the usefulness of microfluidics-based electrochemical biosensors. At present, affordable bioreceptor components acquired from organic constituents like microorganisms and plants are striking potential candidates. However, existing edge design approaches have limitations to the repeatability and stability of analytical outcomes. Herein, incorporating 3D printing, inkjet printing, and screen-printing technology for electrode development will gradually enhance the biosensor’s accuracy, stability, and reproducibility. Furthermore, integrating artificial intelligence, the cyber-physical system, and machine learning will significantly augment the data access and storage unit. Notably, high compassion has always been the most excellent conspicuous property of microfluidic electrochemical detection over other analytical approaches. Furthermore, amalgamated with novel nanomaterials, micro-machining technology could considerably enhance the device’s sensitivity.

**Figure 9:**
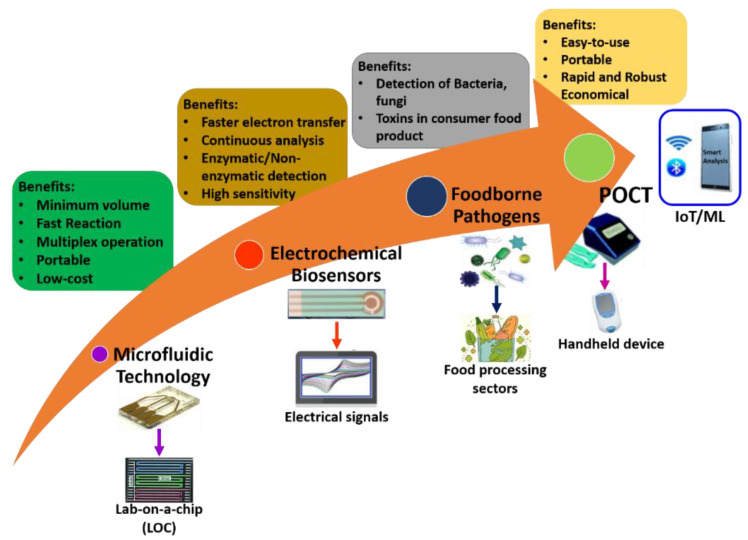
Future directions of microfluidic electrochemical biosensors within sustainable food safety applications.

It is well-known that only some microfluidic electrochemical sensors have been realized, transformed, and commercialized from hypothetical ideas into realistic applications. Miniaturized POCT is a need of the hour for the detection of pathogens and bacteria. Future directions could be a combination of microfluidics, electrochemical sensors, and advanced technology tendencies like artificial intelligence (AI), deep learning, machine learning, and internet-of-things (IoT), and well as solving the misperception of practical applications. Figure 9 illustrates the future directions of microfluidic electrochemical sensors within sustainable food safety applications. Finally, the hope from researchers and scientists in the future is to offer a traverse to link the cleft between microfluidics-based electrochemical sensors and food science.

## Figures and Tables

**Table 1:** Summary of different techniques of electrochemical biosensors.

Transducer	Technique	Merits	Demerits	Ref
Electrochemical	Potentiometric	Possibility of constant study on diverse biomolecule Real-time detection	Time-consuming Profound to the surrounding atmosphere Interference induces by nonspecific binding	[87,88]
	Impedimetric	Instantaneous detection Simplicity User friendly	Requires bulky device Need theoretical simulation for facts study Low sensitivity	[89,90,91]
	Conductometric	Suitable for colored analytes No need for an indicator Accurate	Nonspecificity High interference Increased levels of salt may lead to errors	
	Amperometric	Simple Affordable Miniaturize Easy to operate	Require redox element to boost the production of current Time-consuming	[92,93]

**Table 2:** Summary of electrochemical biosensors for various foodborne pathogens.

Detection Technique	Revised Electrode	Linear Range (CFU/mL)	Bioreceptor Element	Detection Limit	Analyte	Ref.
EIS	ITO/MWCNT/PEI	1–10^4^	Antibody	1 CFU/mL	*E. coli* O157:H7	[93]
EIS & SPR	Au IDE µelectrodes	10^3^–10^6^	Antibody	10^3^ CFU/mL	*E. coli* K12	[98]
Amperometric	PB-altered SPIMs	10–10^6^	Enzyme	10^2^ CFU/mL	*E. coli* O157:H7	[99]
EIS	IDE µelectrode	10–10^5^	Antibody and Aptamer	12 CFU/mL	*E. coli* O157:H7	[100]
EIS	3D-IDEA	10–10^5^	Aptamer	2.8 × 10^2^ CFU/mL	*E. coli* O157:H7	[101]
Amperometric	Au chip	10–3.97 × 10^7^	Antibody	50 CFU/mL	*E. coli*	[102]
EIS	MNPs-Ag/SPIDE	1–10^6^	Melittin	1 CFU/mL	*E. coli*	[103]
EIS	Bridged rebar graphene	10–10^6^	Aptamer	10 CFU/mL	*E. coli* O78:K80:H11	[104]
EIS	NPG/GCE	6.5 × 10^2^–6.5 × 10^8^	Aptamer	1 CFU/mL	*S. typhi*	[105]
Potentiometric	ssDNA/MWCNT/ITO	67–6.7 × 10^5^	Aptamer	10 CFU/mL	*S. typhi*	[106]
DPV	Electrodes array	10–10^2^	Antibody	7.7 CFU/mL	*S. typhi*	[107]
Chronoamperometry	Antibody/protein A/ Au electrode	10–10^6^	Antibody	10 CFU/mL	*S. typhi*	[108]
DPV	Antibody/magnetic beads	Antibody	10–10^7^	3 CFU/mL	*S. typhi*	[109]
EIS	Mannose/Au electrode	Mannose	50–10^3^	50 CFU/mL	*Salmonella ATC14028*	[110]
EIS	SAM/Au-SPEs	Antibody	10^3^–10^7^	-	*S. typhi*	[111]
EIS	Nisin/Au electrode	Nisin	15–1.5 × 10^4^	15 CFU/mL	*S. typhi*	[112]
EIS	Antibody/laser-induced graphene electrode	Antibody	25–10^5^	13 CFU/mL	*S. enterica*	[113]
DPV	SWCNT conjugate/CPE	10–10^7^	Antibody	13 CFU/mL	*S. aureus*	[114]
EIS	TSP/Au electrode	-	Nucleic acid	57 fM	mecA gene	[115]
DPV	Antibody-ALP/anti-PBP2a MNPs/Au electrode	10^3^–10^5^	Antibody	845 CFU/mL	MRSA	[116]
EIS	Antibody/AuNPs/GCE	10–10^7^	Antibody	3.3 CFU/mL	*S. aureus*	[117]
EIS	AgNPs/3D-ZnO/electrode	-	Vancomycin	330 CFU/mL	*S. aureus*	[118]
SWV	A mercury drop electrode in the air	4 × 10^7^–2 × 10^4^	Antibody	2 × 10^4^ CFU/mL	MRSA	[119]
DPV	Triple-helix molecular switch/Au electrode	30–3 × 10^8^	Aptamer	8 CFU/mL	*S. aureus*	[120]
DPV	Phage/PEI/c-MWCNTs/electrode	-	Phage	3 CFU/mL	*S. aureus*	[121]
EIS	IDE array based electrode	1.6 × 10^2^–1.6 × 10^5^	Antibody	1.6 × 10^2^ CFU/mL	Lm	[122]
DPV	ssDNA/RGO/AuNPs/CILE	10^–13^–10^–6^ M	ssDNA	3.17 × 10^–14^ M	Lm	[123]
EIS	MNP(MAb)-Lm-AuNPs (urease- PAb)/SPIE	1.9 × 10^3^–1.9 × 10^6^	Polyclonal antibody	1.6 × 10^3^ CFU/mL	Lm	[124]
Amperometric	AAO/Au electrode	100–1250	Aptamer	10^2^ CFU/mL	Lm	[125]
LSV	Ag+/ALP-secondary antibody/SPCE	-	Antibody	1.5 ng/mL	Lm p60 proteins	[126]
ECL	Cellulose paper electrode	-	DNA	10 copies/µL	Lm	[127]
EIS	IDE Au	2.2 × 10^3^–10^2^	Antibody	5.5 CFU/mL	Lm	[128]
Amperometric	H_2_O_2_/HRP-antibody/MWCNT fibers electrode	10^2^–10^5^	Antibody	1.07 × 10^2^ CFU/mL	Lm	[129]
Amperometric	Pt electrode	10^2^–10^8^	Ferric ammonium citrate and esculin	-	Lm	[130]
SWV	Peptide magnetic/AuNPs/SPCE	-	Peptide	9 CFU/mL	Lm	[131]
DPV	cDNA/AuNPs-DNA/RCA/ aptamer/Antibody/Au electrode	2.2–2.2 × 10^8^	Antibody and aptamer	2 CFU/mL	Vp	[132]
ECL-ASV	Ru-AgNPs@GO-dual antibody/GCE	10^2^–10^7^	Antibody	33 CFU/mL	Vp	[133]
EIS	Cells/antibody/protein A/ APTS-CeO_2_ NWs/ electrode	10^2^–10^7^	Protein A-arbitrated antibody	10^2^ CFU/mL	Vibrio cholerae O1	[134]
EIS	Aptamer/AuNPs/GCE	10–10^6^	Aptamer	1 CFU/mL	Vp	[135]
Amperometric	H_2_O_2_/DNAzymehemin/ G-quadruplex complementary sequences/ SPCE	2.4 × 10^7^–3.84 × 10^4^	Aptamer	5.01 × 10^2^ CFU/mL	Melissococcus Plutonius	[136]
DPV	cDNA/ssDNA probe/ polylactide/AuNPs/SPCE	2.0 × 10^–8^–2.0 × 10^–13^ M	ssDNA	2.16 pM	Vp	[137]
Amperometric	HRP-antibody/Au SPEs/GCE	10^5^–10^9^	Antibody	6.6 × 10^4^ CFU/mL	Melissococcus Plutonius	[138]
DPV	ALP-antibody2/antibody2/Au electrode	-	Antibody	10^2^ CFU/mL	Cholera toxin subunit B	[139]

EIS = electrochemical impedance spectroscopy; CV = cyclic voltammetry; DPV = differential pulse voltammetry; SWV = square wave voltammetry; LSV = linear sweep voltammetry; LAPS = light addressable potentiometric sensor; ECL = electrochemiluminescence; SPR = electrochemical surface plasmon resonance; DNA = deoxyribonucleic acid; *Listeria monocytogenes* (Lm); *Escherichia coli* (*E. coli*), *Salmonella*, *Staphylococcus aureus* (*S. aureus*); *Salmonella typhi* (*S. typhi*).

## Data Availability

Not applicable.

## References

[1] Rivet C., Lee H., Hirsch A., Hamilton S., Lu H. (2011). Microfluidics for medical diagnostics and biosensors. Chem. Eng. Sci..

[2] Zhang Y., Ozdemir P. (2009). Microfluidic DNA amplification—A review. Anal. Chim. Acta.

[3] Bruijns B., van Asten A., Tiggelaar R., Gardeniers H. (2016). Microfluidic devices for forensic DNA analysis: A review. Biosensors.

[4] Pumera M., Merkoçi A., Alegret S. (2006). New materials for electrochemical sensing VII. Microfluidic chip platforms. TrAC Trends Anal. Chem..

[5] Puneeth S.B., Kulkarni M.B., Goel S. (2021). Microfluidic viscometers for biochemical and biomedical applications: A review. Eng. Res. Express.

[6] Velmurugan K., Kulkarni M.B., Gupta I., Das R., Goel S., Nirmal J., Mohanan P.V. (2022). Role of Microfluidics in Drug Delivery. Microfluidics and Multi Organs on Chip.

[7] Davidson E.M., Croal B.L. (2003). Introduction of an Albumin-to-Creatinine Ratio Point-of-Care Device: Analytic, Clinical, and Cost-effectiveness Aspects. Point Care.

[8] Kukkar D., Zhang D., Jeon B.H., Kim K.-H. (2022). Recent advances in wearable biosensors for non-invasive monitoring of specific metabolites and electrolytes associated with chronic kidney disease: Performance evaluation and future challenges. TrAC Trends Anal. Chem..

[9] Asaka S., Yoshizawa A., Matsuda K., Yamaguchi A., Yamamoto H., Shiina T., Nakata R., Ogawa K., Zhang M., Honda T. (2017). A novel, rapid point-of-care test for lung cancer patients to detect epidermal growth factor receptor gene mutations by using real-time droplet-PCR and fresh liquid cytology specimens. Oncol. Rep..

[10] Sciancalepore A.G., Polini A., Mele E., Girardo S., Cingolani R., Pisignano D. (2011). Rapid nested-PCR for tyrosinase gene detection on chip. Biosens. Bioelectron..

[11] Kulkarni M.B., Goel S. (2022). Recent advancements in integrated microthermofluidic systems for biochemical and biomedical applications—A review. Sens. Actuators A Phys..

[12] Kulkarni M.B., Goyal S., Dhar A., Sriram D., Goel S. (2022). Miniaturized and IoT Enabled Continuous-Flow-Based Microfluidic PCR Device for DNA Amplification. IEEE Trans. Nanobiosci..

[13] Kulkarni M.B., Goel S. (2021). Miniaturized DNA amplification platform with soft-lithographically fabricated continuous-flow PCR microfluidic device on a portable temperature controller. Microfluid. Nanofluidics.

[14] Kulkarni M.B., Goel S. (2020). Advances in continuous-flow based microfluidic PCR devices—A review. Eng. Res. Express.

[15] Gou T., Hu J., Wu W., Ding X., Zhou S., Fang W., Mu Y. (2018). Smartphone-based mobile digital PCR device for DNA quantitative analysis with high accuracy. Biosens. Bioelectron..

[16] Dutta G., Rainbow J., Zupancic U., Papamatthaiou S., Estrela P. (2018). Microfluidic Devices for Label-Free DNA Detection. Chemosensors.

[17] Zhang C., Xing D., Li Y. (2007). Micropumps, microvalves, and micromixers within PCR microfluidic chips: Advances and trends. Biotechnol. Adv..

[18] Bhaiyya M., Kulkarni M.B., Pattnaik P.K., Goel S. (2022). Internet of things-enabled photomultiplier tube- and smartphone-based electrochemiluminescence platform to detect choline and dopamine using 3D-printed closed bipolar electrodes. Luminescence.

[19] Kulkarni M.B., Yashas, Enaganti P.K., Amreen K., Goel S. (2020). Internet of Things enabled portable thermal management system with microfluidic platform to synthesize MnO_2_ nanoparticles for electrochemical sensing. Nanotechnology.

[20] Erickson D., Li D. (2004). Integrated microfluidic devices. Anal. Chim. Acta.

[21] Manz A., Harrison D.J., Verpoorte E.M.J., Fettinger J.C., Paulus A., Lüdi H., Widmer H.M. (1992). Planar chips technology for miniaturization and integration of separation techniques into monitoring systems: Capillary electrophoresis on a chip. J. Chromatogr. A.

[22] Kulkarni M.B., Goel S. (2020). Microfluidic devices for synthesizing nanomaterials—A review. Nano Express.

[23] Fair R.B., Khlystov A., Tailor T.D., Ivanov V., Evans R.D., Srinivasan V., Pamula V.K., Pollack M.G., Griffin P.B., Zhou J. (2007). Chemical and biological applications of digital-microfluidic devices. IEEE Des. Test Comput..

[24] Kulkarni M.B., Goel S. (2021). A Review on Recent Advancements in Chamber-Based Microfluidic PCR Devices. Microelectronics and Signal Processing.

[25] Dmytryshyn B. Microfluidic cell culture systems and cellular analysis. Proceedings of the 2011 7th International Conference on Perspective Technologies and Methods in MEMS Design, MEMSTECH 2011.

[26] Kulkarni M.B., Salve M., Goel S. (2021). Miniaturized Thermal Monitoring Module with CO_2_ Laser Ablated Microfluidic Device for Electrochemically Validated DNA Amplification. IEEE Trans. Instrum. Meas..

[27] Currin S.D., Gondwe M.S., Mayindi N.B., Chipungu S., Khoza B.L., Tollman S., Fabian J., George J.A. (2021). Diagnostic accuracy of semiquantitative point of care urine albumin to creatinine ratio and urine dipstick analysis in a primary care resource limited setting in South Africa. BMC Nephrol..

[28] Soni A., Kumar R., Kumar S. (2018). Chemical Smartphone based optical biosensor for the detection of urea in saliva. Sens. Actuators B Chem..

[29] Ding X., Srinivasan B., Tung S. (2015). Development and Applications of Portable Biosensors. J. Lab. Autom..

[30] Zhang L., Ding B., Chen Q., Feng Q., Lin L., Sun J. (2017). Point-of-care-testing of nucleic acids by microfluidics. TrAC Trends Anal. Chem..

[31] Wang D., Chan H.N., Liu Z., Micheal S., Li L., Baniani D.B., Tan M.J.A., Huang L., Wang J. (2020). Recent Developments in Microfluidic-Based Point-of-care Testing (POCT) Diagnoses. Nanotechnology and Microfluidics.

[32] Alsaba M.T., Al M.F., Ahmed D. (2020). A comprehensive review of nanoparticles applications in the oil and gas industry. J. Pet. Explor. Prod. Technol..

[33] Kulkarni M.B., Ayachit N.H., Aminabhavi T.M. (2022). Biosensors and Microfluidic Biosensors: From Fabrication to Application. Biosensors.

[34] Chou J.C., Wu C.Y., Kuo P.Y., Lai C.H., Nien Y.H., Wu Y.X., Lin S.H., Liao Y.H. (2019). The Flexible Urea Biosensor Using Magnetic Nanoparticles. IEEE Trans. Nanotechnol..

[35] Roy A., Ray A., Saha S., Ghosh M., Das T., Satpati B., Nandi M., Das S. (2018). NiO-CNT composite for high performance supercapacitor electrode and oxygen evolution reaction. Electrochim. Acta.

[36] Chen J., Meng H., Tian Y., Yang R., Du D., Li Z., Qu L., Lin Y. (2019). Recent advances in functionalized MnO_2_ nanosheets for biosensing and biomedicine applications. Nanoscale Horiz..

[37] Pal A., Kulkarni M.B., Gupta H., Ponnalagu R.N., Dubey S.K., Goel S. (2021). Portable and Autonomous Device for Real-time Colorimetric Detection: Validation for Phosphorous and Nitrite Detection. Sens. Actuators A Phys..

[38] Srikanth S., Dudala S., Jayapiriya U.S., Mohan J.M., Raut S., Dubey S.K., Ishii I., Javed A., Goel S. (2021). Droplet-based lab-on-chip platform integrated with laser ablated graphene heaters to synthesize gold nanoparticles for electrochemical sensing and fuel cell applications. Sci. Rep..

[39] Thakur M., Wang B., Verma M.L. (2022). Development and applications of nanobiosensors for sustainable agricultural and food industries: Recent developments, challenges and perspectives. Environ. Technol. Innov..

[40] Kulkarni M.B., Channappa Bhyri D., Vanjerkhede K. (2018). Brain Tumor Detection using Random Walk Solver Based Segmentation from MRI. Microsc. Res. Tech..

[41] Holzinger M., Le Goff A., Cosnier S. (2014). Nanomaterials for biosensing applications: A review. Front. Chem..

[42] Fernández-Carballo B.L., McBeth C., McGuiness I., Kalashnikov M., Baum C., Borrós S., Sharon A., Sauer-Budge A.F. (2018). Continuous-flow, microfluidic, qRT-PCR system for RNA virus detection. Anal. Bioanal. Chem..

[43] Skottrup P.D., Nicolaisen M., Justesen A.F. (2008). Towards on-site pathogen detection using antibody-based sensors. Biosens. Bioelectron..

[44] Khaliliazar S., Ouyang L., Piper A., Chondrogiannis G., Hanze M., Herland A., Herland A., Hamedi M.M. (2020). Electrochemical Detection of Genomic DNA Utilizing Recombinase Polymerase Amplification and Stem-Loop Probe. ACS Omega.

[45] Kulkarni M.B., Ayachit N.H., Aminabhavi T.M. (2022). Recent Advancements in Nanobiosensors: Current Trends, Challenges, Applications, and Future Scope. Biosensors.

[46] Pérez-Fernández B., de la Escosura-Muñiz A. (2022). Electrochemical biosensors based on nanomaterials for aflatoxins detection: A review (2015–2021). Anal. Chim. Acta.

[47] Sheen H.-J., Panigrahi B., Kuo T.-R., Hsu W.-C., Chung P.-S., Xie Q.-Z., Lin C.-Y., Chang Y.-S., Lin C.-T., Fan Y.-J. (2022). Electrochemical biosensor with electrokinetics-assisted molecular trapping for enhancing C-reactive protein detection. Biosens. Bioelectron..

[48] Lin C.-Y., Nhat Nguyen U.T., Hsieh H.-Y., Tahara H., Chang Y.-S., Wang B.-Y., Gu B.-C., Dai Y.-H., Wu C.-C., Tsai I.-J. (2022). Peptide-based electrochemical sensor with nanogold enhancement for detecting rheumatoid arthritis. Talanta.

[49] Reddy Y.V.M., Shin J.H., Palakollu V.N., Sravani B., Choi C.-H., Park K., Kim S.-K., Madhavi G., Park J.P., Shetti N.P. (2022). Strategies, advances, and challenges associated with the use of graphene-based nanocomposites for electrochemical biosensors. Adv. Colloid Interface Sci..

[50] Kampeera J., Pasakon P., Karuwan C., Arunrut N., Sappat A., Sirithammajak S., Dechokiattawan N., Sumranwanich T., Chaivisuthangkura P., Ounjai P. (2019). Point-of-care rapid detection of Vibrio parahaemolyticus in seafood using loop-mediated isothermal amplification and graphene-based screen-printed electrochemical sensor. Biosens. Bioelectron..

[51] Ren R., Lu D., Liu T. (2021). Development of a sandwich-type rat small intestine tissue sensor for detecting resveratrol and its receptors. Biomed. Microdevices.

[52] Kulkarni M.B. (2016). Detection of Brain Tumor Using K-Means Clustering. Int. J. Sci. Res..

[53] Ankri S., Mirelman D. (1999). Antimicrobial properties of allicin from garlic. Microbes Infect..

[54] Gui Q., Lawson T., Shan S., Yan L., Liu Y. (2017). The application of whole cell-based biosensors for use in environmental analysis and in medical diagnostics. Sensors.

[55] Aquino A., Conte-Junior C.A. (2020). A Systematic Review of Food Allergy: Nanobiosensor and Food Allergen Detection. Biosensors.

[56] Leong D., Alvarez-Ordóñez A., Jordan K. (2014). Monitoring occurrence and persistence of *Listeria monocytogenes* in foods and food processing environments in the Republic of Ireland. Front. Microbiol..

[57] Alava T., Berthet-Duroure N., Ayela C., Trévisiol E., Pugnière M., Morel Y., Rameil P., Nicu L. (2009). Parallel acoustic detection of biological warfare agents surrogates by means of piezoelectric immunochips. Sens. Actuators B Chem..

[58] Oh S.J., Park B.H., Choi G., Seo J.H., Jung J.H., Choi J.S., Kim D.H., Seo T.S. (2016). Fully automated and colorimetric foodborne pathogen detection on an integrated centrifugal microfluidic device. Lab Chip.

[59] Pandey P., Merwyn S., Agarwal G.S., Tripathi B.K., Pant S.C. (2012). Electrochemical synthesis of multi-armed CuO nanoparticles and their remarkable bactericidal potential against waterborne bacteria. J. Nanoparticle Res..

[60] Beno S.M., Stasiewicz M.J., Andrus A.D., Ralyea R.D., Kent D.J., Martin N.H., Wiedmann M., Boor K.J. (2016). Development and validation of pathogen environmental monitoring programs for small cheese processing facilities. J. Food Prot..

[61] Kant K., Shahbazi M.A., Dave V.P., Ngo T.A., Chidambara V.A., Than L.Q., Bang D.D., Wolff A. (2018). Microfluidic devices for sample preparation and rapid detection of foodborne pathogens. Biotechnol. Adv..

[62] Kuang H., Zhao Y., Ma W., Xu L., Wang L., Xu C. (2011). Recent developments in analytical applications of quantum dots. TrAC Trends Anal. Chem..

[63] Hertneky B., Eger J., Bailly M., Christen J.B. Mobile and Efficient Temperature and Humidity Control Chamber for Point-of-Care Diagnostics. Proceedings of the 2019 IEEE Healthcare Innovations and Point of Care Technologies, HI-POCT 2019.

[64] Si H., Xu G., Jing F., Sun P., Zhao D., Wu D. (2020). A multi-volume microfluidic device with no reagent loss for low-cost digital PCR application. Sens. Actuators B Chem..

[65] Soy S., Sharma S.R., Nigam V.K. (2022). Bio-fabrication of thermozyme-based nano-biosensors: Their components and present scenario. J. Mater. Sci. Mater. Electron..

[66] Bhatt G., Bhattacharya S. (2019). Biosensors on chip: A critical review from an aspect of micro/nanoscales. J. Micromanufacturing.

[67] Azizipour N., Avazpour R., Rosenzweig D.H., Sawan M., Ajji A. (2020). Evolution of biochip technology: A review from lab-on-a-chip to organ-on-a-chip. Micromachines.

[68] Kulkarni M.B., Velmurugan K., Prasanth E., Amreen K., Nirmal J., Goel S. (2021). Smartphone enabled miniaturized temperature controller platform to synthesize nio/cuo nanoparticles for electrochemical sensing and nanomicelles for ocular drug delivery applications. Biomed. Microdevices.

[69] Samiei E., Tabrizian M., Hoorfar M. (2016). A review of digital microfluidics as portable platforms for lab-on a-chip applications. Lab Chip.

[70] Wang Z.L. (2013). Triboelectric nanogenerators as new energy technology for self-powered systems and as active mechanical and chemical sensors. ACS Nano.

[71] Lee T.Y., Han K., Barrett D.O., Park S., Soper S.A., Murphy M.C. (2018). Accurate, predictable, repeatable micro-assembly technology for polymer, microfluidic modules. Sens. Actuators B Chem..

[72] Maduraiveeran G., Chen A. (2021). Design of an enzyme-mimicking NiO@Au nanocomposite for the sensitive electrochemical detection of lactic acid in human serum and urine. Electrochim. Acta.

[73] Stobiecka A., Radecka H., Radecki J. (2007). Novel voltammetric biosensor for determining acrylamide in food samples. Biosens. Bioelectron..

[74] Nath D., Sai Kiran P., Rewatkar P., Krishnamurthy B., Sankar Ganesh P., Goel S. (2019). Escherichia coli Fed Paper-Based Microfluidic Microbial Fuel Cell with MWCNT Composed Bucky Paper Bioelectrodes. IEEE Trans. Nanobiosci..

[75] Salve M., Amreen K., Pattnaik P.K., Goel S. (2020). Miniaturized Platform with Nanocomposite Optimized Pencil Electrodes for Selective Non-Interfering Electrochemical Sensing. IEEE Trans. Nanotechnol..

[76] Bandapati M., Krishnamurthy B., Goel S. (2019). Fully assembled membraneless glucose biofuel cell with MWCNT modified pencil graphite leads as novel bioelectrodes. IEEE Trans. Nanobiosci..

[77] Bandapati M., Goel S., Krishnamurthy B. (2019). Platinum utilization in proton exchange membrane fuel cell and direct methanol fuel cell—Review. J. Electrochem. Sci. Eng..

[78] Mohan J.M., Amreen K., Javed A., Dubey S.K., Goel S. (2020). Modified Graphite Paper Based Miniaturized Electrochemically Optimized Hydrazine Sensing Platform. ECS J. Solid State Sci. Technol..

[79] Amreen K., Nisha S., Senthil Kumar A. (2018). Undiluted human whole blood uric acid detection using a graphitized mesoporous carbon modified electrode: A potential tool for clinical point-of-care uric acid diagnosis. Analyst.

[80] Chen Y.-S., Huang C.-H., Pai P.-C., Seo J., Lei K.F. (2023). A Review on Microfluidics-Based Impedance Biosensors. Biosensors.

[81] Kashyap D., Yadav R.S., Gohil S., Venkateswaran P.S., Pandey J.K., Kim G.M., Kim Y.H., Dwivedi P.K., Sharma A., Ayyub P. (2015). Fabrication of vertically aligned copper nanotubes as a novel electrode for enzymatic biofuel cells. Electrochim. Acta.

[82] Kesavan G., Nataraj N., Chen S.M., Lin L.H. (2020). Hydrothermal synthesis of NiFe_2_O_4_ nanoparticles as an efficient electrocatalyst for the electrochemical detection of bisphenol A. New J. Chem..

[83] Röhlen D.L., Pilas J., Dahmen M., Keusgen M., Selmer T., Schöning M.J. (2018). Toward a hybrid biosensor system for analysis of organic and volatile fatty acids in fermentation processes. Front. Chem..

[84] Shu Y., Yan Y., Chen J., Xu Q., Pang H., Hu X. (2017). Ni and NiO Nanoparticles Decorated Metal-Organic Framework Nanosheets: Facile Synthesis and High-Performance Nonenzymatic Glucose Detection in Human Serum. ACS Appl. Mater. Interfaces.

[85] Holonyak N., Bevacqua S.F. (1962). Coherent (visible) light emission from Ga(As_1−*x*_P*_x_*) junctions. Appl. Phys. Lett..

[86] Sung W.J., Bae Y.H. (2006). Glucose oxidase, lactate oxidase, and galactose oxidase enzyme electrode based on polypyrrole with polyanion/PEG/enzyme conjugate dopant. Sens. Actuators B Chem..

[87] Karimi-Maleh H., Orooji Y., Karimi F., Alizadeh M., Baghayeri M., Rouhi J., Tajik S., Beitollahi H., Agarwal S., Gupta V.K. (2021). A critical review on the use of potentiometric based biosensors for biomarkers detection. Biosens. Bioelectron..

[88] Shaibani P.M., Etayash H., Jiang K., Sohrabi A., Hassanpourfard M., Naicker S., Sadrzadeh M., Thundat T. (2018). Portable Nanofiber-Light Addressable Potentiometric Sensor for Rapid Escherichia coli Detection in Orange Juice. ACS Sens..

[89] Chuang Y.H., Chang Y.T., Liu K.L., Chang H.Y., Yew T.R. (2011). Electrical impedimetric biosensors for liver function detection. Biosens. Bioelectron..

[90] Mishra A., Pilloton R., Jain S., Roy S., Khanuja M., Mathur A., Narang J. (2022). Paper-Based Electrodes Conjugated with Tungsten Disulfide Nanostructure and Aptamer for Impedimetric Detection of Listeria monocytogenes. Biosensors.

[91] Rushworth J.V., Ahmed A., Griffiths H.H., Pollock N.M., Hooper N.M., Millner P.A. (2014). A label-free electrical impedimetric biosensor for the specific detection of Alzheimer’s amyloid-beta oligomers. Biosens. Bioelectron..

[92] Yang Q., Li N., Li Q., Chen S., Wang H.L., Yang H. (2019). Amperometric sarcosine biosensor based on hollow magnetic Pt–Fe_3_O_4_@C nanospheres. Anal. Chim. Acta.

[93] Wu Z.L., Li C.K., Yu J.G., Chen X.Q. (2017). MnO_2_/reduced graphene oxide nanoribbons: Facile hydrothermal preparation and their application in amperometric detection of hydrogen peroxide. Sens. Actuators B Chem..

[94] Wang K., Lin X., Zhang M., Li Y., Luo C., Wu J. (2022). Review of Electrochemical Biosensors for Food Safety Detection. Biosensors.

[95] Review A.U. (2022). Electrochemical Biosensors for Pathogen Detection: An Updated Review. Biosensors.

[96] Dutta P., Lu Y.-J., Hsieh H.-Y., Lee T.-Y., Lee Y.-T., Cheng C.-M., Fan Y.-J. (2021). Detection of Candida albicans Using a Manufactured Electrochemical Sensor. Micromachines.

[97] Fan Y.-J., Hsu Y.-C., Gu B.-C., Wu C.-C. (2020). Voltammetric measurement of Escherichia coli concentration through p-APG hydrolysis by endogenous β-galactosidase. Microchem. J..

[98] Helali S., Sawelem Eid Alatawi A., Abdelghani A. (2018). Pathogenic Escherichia coli biosensor detection on chicken food samples. J. Food Saf..

[99] Xu M., Wang R., Li Y. (2016). An electrochemical biosensor for rapid detection of: *E. coli* O157:H7 with highly efficient bi-functional glucose oxidase-polydopamine nanocomposites and Prussian blue modified screen-printed interdigitated electrodes. Analyst.

[100] Yao L., Wang L., Huang F., Cai G., Xi X., Lin J. (2018). A microfluidic impedance biosensor based on immunomagnetic separation and urease catalysis for continuous-flow detection of *E. coli* O157:H7. Sens. Actuators B Chem..

[101] Brosel-Oliu S., Ferreira R., Uria N., Abramova N., Gargallo R., Muñoz-Pascual F.X., Bratov A. (2018). Novel impedimetric aptasensor for label-free detection of *Escherichia coli* O157:H7. Sens. Actuators B Chem..

[102] Altintas Z., Akgun M., Kokturk G., Uludag Y. (2018). A fully automated microfluidic-based electrochemical sensor for real-time bacteria detection. Biosens. Bioelectron..

[103] Wilson D., Materón E., Ibáñez-Redín G., Faria R.C., Correa D.S., Oliveira O.N. (2019). Erratum to “Electrical detection of pathogenic bacteria in food samples using information visualization methods with a sensor based on magnetic nanoparticles functionalized with antimicrobial peptides”. Talanta.

[104] Kaur H., Shorie M., Sharma M., Ganguli A.K., Sabherwal P. (2017). Bridged Rebar Graphene functionalized aptasensor for pathogenic *E. coli* O78:K80:H11 detection. Biosens. Bioelectron..

[105] Ranjbar S., Shahrokhian S., Nurmohammadi F. (2018). Nanoporous gold as a suitable substrate for preparation of a new sensitive electrochemical aptasensor for detection of *Salmonella typhimurium*. Sens. Actuators B Chem..

[106] Hasan M.R., Pulingam T., Appaturi J.N., Zifruddin A.N., Teh S.J., Lim T.W., Ibrahim F., Leo B.F., Thong K.L. (2018). Carbon nanotube-based aptasensor for sensitive electrochemical detection of whole-cell *Salmonella*. Anal. Biochem..

[107] de Oliveira T.R., Martucci D.H., Faria R.C. (2018). Simple disposable microfluidic device for *Salmonella typhimurium* detection by magneto-immunoassay. Sens. Actuators B Chem..

[108] Melo A.M.A., Alexandre D.L., Oliveira M.R.F., Furtado R.F., Borges M.F., Ribeiro P.R.V., Biswas A., Cheng H.N., Alves C.R., Figueiredo E.A.T. (2018). Optimization and characterization of a biosensor assembly for detection of *Salmonella* Typhimurium. J. Solid State Electrochem..

[109] Bu S.-J., Wang K.-Y., Liu X., Ma L., Wei H.-G., Zhang W.-G., Liu W.-S., Wan J.-Y. (2020). Ferrocene-functionalized nanocomposites as signal amplification probes for electrochemical immunoassay of *Salmonella typhimurium*. Microchim. Acta.

[110] Cui F., Xu Y., Wang R., Liu H., Chen L., Zhang Q., Mu X. (2018). Label-free impedimetric glycan biosensor for quantitative evaluation interactions between pathogenic bacteria and mannose. Biosens. Bioelectron..

[111] Pagliarini V., Neagu D., Scognamiglio V., Pascale S., Scordo G., Volpe G., Delibato E., Pucci E., Notargiacomo A., Pea M. (2019). Treated Gold Screen-Printed Electrode as Disposable Platform for Label-Free Immunosensing of *Salmonella* Typhimurium. Electrocatalysis.

[112] Malvano F., Pilloton R., Albanese D. (2020). A novel impedimetric biosensor based on the antimicrobial activity of the peptide nisin for the detection of *Salmonella* spp.. Food Chem..

[113] Soares R.R.A., Hjort R.G., Pola C.C., Parate K., Reis E.L., Soares N.F.F., McLamore E.S., Claussen J.C., Gomes C.L. (2020). Laser-Induced Graphene Electrochemical Immunosensors for Rapid and Label-Free Monitoring of *Salmonella enterica* in Chicken Broth. ACS Sens..

[114] Bhardwaj J., Devarakonda S., Kumar S., Jang J. (2017). Development of a paper-based electrochemical immunosensor using an antibody-single walled carbon nanotubes bio-conjugate modified electrode for label-free detection of foodborne pathogens. Sens. Actuators B Chem..

[115] Xu L., Liang W., Wen Y., Wang L., Yang X., Ren S., Jia N., Zuo X., Liu G. (2018). An ultrasensitive electrochemical biosensor for the detection of mecA gene in methicillin-resistant Staphylococcus aureus. Biosens. Bioelectron..

[116] Nemr C.R., Smith S.J., Liu W., Mepham A.H., Mohamadi R.M., Labib M., Kelley S.O. (2019). Nanoparticle-Mediated Capture and Electrochemical Detection of Methicillin-Resistant *Staphylococcus aureus*. Anal. Chem..

[117] Roushani M., Rahmati Z., Golchin M., Lotfi Z., Nemati M. (2020). Electrochemical immunosensor for determination of *Staphylococcus aureus* bacteria by IgY immobilized on glassy carbon electrode with electrodeposited gold nanoparticles. Microchim. Acta.

[118] Yang Z., Wang Y., Zhang D. (2017). A novel multifunctional electrochemical platform for simultaneous detection, elimination, and inactivation of pathogenic bacteria based on the Vancomycin-functionalised AgNPs/3D-ZnO nanorod arrays. Biosens. Bioelectron..

[119] Cihalova K., Hegerova D., Dostalova S., Jelinkova P., Krejcova L., Milosavljevic V., Krizkova S., Kopel P., Adam V. (2016). Particle-based immunochemical separation of methicillin resistant *Staphylococcus aureus* with indirect electrochemical detection of labeling oligonucleotides. Anal. Methods.

[120] Cai R., Zhang Z., Chen H., Tian Y., Zhou N. (2021). A versatile signal-on electrochemical biosensor for *Staphylococcus aureus* based on triple-helix molecular switch. Sens. Actuators B Chem..

[121] Farooq U., Ullah M.W., Yang Q., Aziz A., Xu J., Zhou L., Wang S. (2020). High-density phage particles immobilization in surface-modified bacterial cellulose for ultra-sensitive and selective electrochemical detection of *Staphylococcus aureus*. Biosens. Bioelectron..

[122] Chen Q., Wang D., Cai G., Xiong Y., Li Y., Wang M., Huo H., Lin J. (2016). Fast and sensitive detection of foodborne pathogen using electrochemical impedance analysis, urease catalysis and microfluidics. Biosens. Bioelectron..

[123] Niu X., Zheng W., Yin C., Weng W., Li G., Sun W., Men Y. (2017). Electrochemical DNA biosensor based on gold nanoparticles and partially reduced graphene oxide modified electrode for the detection of *Listeria monocytogenes* hly gene sequence. J. Electroanal. Chem..

[124] Wang D., Chen Q., Huo H., Bai S., Cai G., Lai W., Lin J. (2017). Efficient separation and quantitative detection of *Listeria monocytogenes* based on screen-printed interdigitated electrode, urease and magnetic nanoparticles. Food Control.

[125] Lu Y., Liu Y., Zhao Y., Li W., Qiu L., Li L. (2016). A Novel and Disposable Enzyme-Labeled Amperometric Immunosensor Based on MWCNT Fibers for *Listeria monocytogenes* Detection. J. Nanomater..

[126] Silva N.F.D., Neves M.M.P.S., Magalhães J.M.C.S., Freire C., Delerue-Matos C. (2020). Electrochemical immunosensor towards invasion-associated protein p60: An alternative strategy for *Listeria monocytogenes* screening in food. Talanta.

[127] Liu H., Zhou X., Liu W., Yang X., Xing D. (2016). Paper-Based Bipolar Electrode Electrochemiluminescence Switch for Label-Free and Sensitive Genetic Detection of Pathogenic Bacteria. Anal. Chem..

[128] Chiriacò M.S., Parlangeli I., Sirsi F., Poltronieri P., Primiceri E. (2018). Impedance Sensing Platform for Detection of the Food Pathogen Listeria monocytogenes. Electronics.

[129] Li Z., Zhang J., Huang Y., Zhai J., Liao G., Wang Z., Ning C. (2022). Development of electroactive materials-based immunosensor towards early-stage cancer detection. Coord. Chem. Rev..

[130] Huang Y.-M., Hsu H.-Y., Hsu C.-L. (2016). Development of electrochemical method to detect bacterial count, *Listeria monocytogenes*, and somatic cell count in raw milk. J. Taiwan Inst. Chem. Eng..

[131] Eissa S., Zourob M. (2020). Ultrasensitive peptide-based multiplexed electrochemical biosensor for the simultaneous detection of *Listeria monocytogenes* and *Staphylococcus aureus*. Microchim. Acta.

[132] Teng J., Ye Y., Yao L., Yan C., Cheng K., Xue F., Pan D., Li B., Chen W. (2017). Rolling circle amplification based amperometric aptamer/immuno hybrid biosensor for ultrasensitive detection of *Vibrio parahaemolyticus*. Microchim. Acta.

[133] Wang T., Song X., Lin H., Hao T., Hu Y., Wang S., Su X., Guo Z. (2019). A Faraday cage-type immunosensor for dual-modal detection of Vibrio parahaemolyticus by electrochemiluminescence and anodic stripping voltammetry. Anal. Chim. Acta.

[134] Tam P.D., Hoang N.L., Lan H., Vuong P.H., Anh T.T.N., Huy T.Q., Thuy N.T. (2016). Detection of vibrio cholerae O1 by using cerium oxide nanowires—Based immunosensor with different antibody immobilization methods. J. Korean Phys. Soc..

[135] Zarei S.S., Soleimanian-Zad S., Ensafi A.A. (2018). An impedimetric aptasensor for *Shigella dysenteriae* using a gold nanoparticle-modified glassy carbon electrode. Microchim. Acta.

[136] Yuan Y., Wu X., Liu Z., Ning Q., Fu L., Wu S. (2020). A signal cascade amplification strategy based on RT-PCR triggering of a G-quadruplex DNAzyme for a novel electrochemical detection of viable *Cronobacter sakazakii*. Analyst.

[137] Nordin N., Yusof N.A., Abdullah J., Radu S., Hushiarian R. (2017). A simple, portable, electrochemical biosensor to screen shellfish for *Vibrio parahaemolyticus*. AMB Express.

[138] Mikušová Z., Farka Z., Pastucha M., Poláchová V., Obořilová R., Skládal P. (2019). Amperometric Immunosensor for Rapid Detection of Honeybee Pathogen Melissococcus Plutonius. Electroanalysis.

[139] Valera A.E., Nesbitt N.T., Archibald M.M., Naughton M.J., Chiles T.C. (2019). On-Chip Electrochemical Detection of Cholera Using a Polypyrrole-Functionalized Dendritic Gold Sensor. ACS Sens..

[140] Buja I., Sabella E., Monteduro A.G., Rizzato S., De Bellis L., Elicio V., Formica L., Luvisi A., Maruccio G. (2022). Detection of Ampelovirus and Nepovirus by Lab-on-a-Chip: A Promising Alternative to ELISA Test for Large Scale Health Screening of Grapevine. Biosensors.

[141] Antonacci A., Arduini F., Attaallah R., Amine A., Giardi M.T., Scognamiglio V. (2022). A Proof-of-Concept Electrochemical Cytosensor Based on *Chlamydomonas reinhardtii* Functionalized Carbon Black Screen-Printed Electrodes: Detection of *Escherichia coli* in Wastewater as a Case Study. Biosensors.

[142] Sidhu R.K., Cavallaro N.D., Pola C.C., Danyluk M.D., Mclamore E.S., Gomes C.L. (2020). Planar Interdigitated Aptasensor for Flow-Through Detection of *Listeria* spp. in Hydroponic Lettuce Growth Media Raminderdeep. Sensors.

[143] Baldini F., Minunni M. (2019). New developments in biosensors. Anal. Bioanal. Chem..

[144] Knauer A., Michael Koehler J. (2020). Screening of nanoparticle properties in microfluidic syntheses. Nanotechnol. Rev..

[145] Rao V.N., Reddy N.L., Kumari M.M., Cheralathan K.K., Ravi P., Sathish M., Neppolian B., Reddy K.R., Shetti N.P., Prathap P. (2019). Sustainable hydrogen production for the greener environment by quantum dots-based efficient photocatalysts: A review. J. Environ. Manag..

[146] Kulkarni M.B., Enaganti P.K., Amreen K., Goel S. (2021). Integrated Temperature Controlling Platform to Synthesize ZnO Nanoparticles and its Deposition on Al-Foil for Biosensing. IEEE Sens. J..

[147] Mohan J.M., Amreen K., Kulkarni M.B., Javed A., Dubey S.K., Goel S. (2021). Optimized Ink Jetted Paper Device for Electroanalytical Detection of Picric Acid. Colloids Surfaces B Biointerfaces.

[148] Eppler R.K., Hudson E.P., Chase S.D., Dordick J.S., Reimer J.A., Clark D.S. (2008). Biocatalyst activity in nonaqueous environments correlates with centisecond-range protein motions. Proc. Natl. Acad. Sci. USA.

[149] Robinson P.K. (2015). Enzymes: Principles and biotechnological applications. Essays Biochem..

[150] Dissanayake M., Vasiljevic T. (2009). Functional properties of whey proteins affected by heat treatment and hydrodynamic high-pressure shearing. J. Dairy Sci..

[151] Nordstrand J., Dutta J. (2019). Dynamic Langmuir Model: A Simpler Approach to Modeling Capacitive Deionization. J. Phys. Chem. C.

[152] Prinz H. (2010). Hill coefficients, dose-response curves and allosteric mechanisms. J. Chem. Biol..

[153] Domanskyi S., Privman V. (2012). Design of digital response in enzyme-based bioanalytical systems for information processing applications. J. Phys. Chem. B.

[154] Grieshaber D., MacKenzie R., Vörös J., Reimhult E. (2008). Electrochemical Biosensors—Sensor Principles and Architectures. Sensors.

[155] Qiu C., Chen X., Rexida R., Shen Y., Qi Q., Bao X., Hou J. (2020). Engineering transcription factor-based biosensors for repressive regulation through transcriptional deactivation design in *Saccharomyces cerevisiae*. Microb. Cell Factories.

[156] Bhalla N., Jolly P., Formisano N., Estrela P. (2016). Introduction to biosensors. Essays Biochem..

[157] Ahmed A., Rushworth J.V., Hirst N.A., Millner P.A. (2014). Biosensors for whole-cell bacterial detection. Clin. Microbiol. Rev..

[158] Pol R., Céspedes F., Gabriel D., Baeza M. (2017). Microfluidic lab-on-a-chip platforms for environmental monitoring. TrAC Trends Anal. Chem..

[159] Meshram B.D., Agrawal A.K., Adil S., Ranvir S., Sande K.K. (2018). Biosensor and its Application in Food and Dairy Industry: A Review. Int. J. Curr. Microbiol. Appl. Sci..

[160] Kulkarni M.B., Us J., Amreen K., Goel S. (2021). Portable Thermal Management Platform for Synthesis of ZnO Nanoparticle in a Microfluidic Device: Validated for Electrochemical Sensing and Glucose Fuel Cell Applications. IEEE Trans. Electron Devices.

[161] Wagner T., Vornholt W., Werner C.F., Yoshinobu T., Miyamoto K., Keusgen M., Schöning M.J. (2016). Light-addressable potentiometric sensor (LAPS) combined with magnetic beads for pharmaceutical screening. Phys. Med..

[162] Nasseri B., Soleimani N., Rabiee N., Kalbasi A., Karimi M., Hamblin M.R. (2018). Point-of-care microfluidic devices for pathogen detection. Biosens. Bioelectron..

[163] Rao C.N.R., Kulkarni G.U., Govindaraj A., Satishkumar B.C., Thomas P.J. (2000). Metal nanoparticles, nanowires, and carbon nanotubes. Pure Appl. Chem..

[164] Kulkarni M.B., Upadhyaya K., Ayachit N.H., Iyer N. (2022). Quantum Dot—Polymer Composites in Light-Emitting Diode Applications. Quantum Dots and Polymer Nanocomposites.

